# Treatment of Fractures by Means of Plaster of Paris

**Published:** 1875-10

**Authors:** 


### Treatment of Fractures by means of the Plaster-of-Paris Bandage.

The results obtained by the use of plaster of Paris as an immovable dressing in cases of fracture of the extremities place it in advance of any of the other dressings of its class. The main disadvantage is its weight; but this is more than compensated for by the readiness with which it may be applied, the quickness with which it solidifies, and its relative strength. It is the intention of the present article to furnish those who have not had the opportunity to learn its use, with the ordinary method of its application as practiced in the New York hospitals, so that they may obtain results equal to those that are there obtained. Differences of opinion exists as to whether it is advisable to put up factures immediately or not, but the majority of conservative surgeons believe that the immediate method exposes the patient to too much risk of sloughing, and possible gangrene. This is particularly true in those cases in which the patient is not under the immediate observation of the surgeon. As a rule, therefore, it is safest and best to allow the limb to rest for a few days, or even a week, so that the swelling shall cease to advance and begin to recede before placing it in plaster. It will be found also that, in a day or two after the fracture has been put in plaster, it will be necessary to cut the plaster down anteriorly by means of a sharp knife. If the splint is then loose, from the further subsidence of the swelling, the sides of it may be brought close to the limb by means of an ordinary roller bandage. When the swelling has been pretty thoroughly reduced, the surgeon will be enabled to put the fracture up permanently Another advantage in inspecting the fracture, particularly if it is of the tibia, is, that a projecting fragment of bone may press against the splint, and in a day or two cause an abrasion, which, if not attended to, by forming a fenestra, would result eventually in a compound fracture.

In order that the subject may be made as clear as possible, it is advisable to divide the contideration of the matter into the following steps:

*The Preparation of the Bandage.*

*Its Mode of Application.*

*The Means of applying it in Special Fractures.*

*The Modification that must be practiced in Compound and some Varieties of Simple Fractures.*

*Its Removal.*

*The Preparation of the Bandage.*—It was formerly, and by many is now the custom to use an ordinary roller-bandage of unbleached muslin as the basis upon which to spread the dry plaster of Paris. Latterly, a marked improvement upon this has been introduced in the shape of a gauze bandage having about twenty threads to the inch. This gauze is used by dress-makers as a stiffening for ladies' dresses, and the only precaution to bear in mind is to get the variety which has not been stiffened with starch. When this bandage has been obtained, it is evenly spread over with plaster of Paris to the depth of a line. The bandage is then rolled very loosely up, for a reason that will appear in the next step.

*The Mode of Application.*—The fracture is first reduced, and the limb is then covered from the extremity with either a layer of cotton-wool, a piece of old blanket, or in fractures of the leg with a long, woolen stocking. The bandage is then soaked in water till it is thoroughly wet, and the time required for this depends mainly upon the lightness with which the bandage is rolled up after being covered with plaster. In the case of the gauze bandage referred to, two minutes will be sufficient, but other varieties may take longer. The limb being thus enveloped with a dry dressing as directed, the wet plaster bandage is commenced at the toes in the lower, and at the palm of the hand in the upper extremities, and carried as far up as may be necessary to go. The thickness of the plaster-dressing will vary with each individual case, but the average is about three folds of the bandage.

*Its Application in Special Fractures; Fractures of the Tibia and Fibula.*—It is only in exceptional cases that extension is required in putting up fractures of the leg. The bandage is commenced at the toes and carried up as far as the head of the tibia, and any deformity that has occured during the manipulation is rectified before the plaster hardens. In exceptional cases, such as fracture of both bones of the leg, extension is required, and to a novice this presents a serious difficulty. It has been met, however. by a device of Dr. Bates, house-surgeon at Bellevue Hospital, and consists in placing one strip of adhesive plaster around the instep, and another around the heel, so arranged that a cord can be attached to the ends of the adhesive plaster, by which extension can be applied. After the requisite amount of extension has been obtained, the plaster of Paris dressing is applied in the manner previously directed; and when it has sufficiently hardened, the ends of the protruding adhesive plaster can be cut off, leaving extension and counter-extension fully maintained by the plaster of Paris.

*Fractures of the Patella.*—Fractures of the patella are treated in two ways: Either the fragments of the patella may be approximated as near as possible by means of adhesive plaster, and then the plaster of Paris bandage may be carried from the toes up to the groin, so as to keep the limb immovable; or the following method may be dopted, which allows of the continual observation of the position of the fragments: A plaster of Paris bandage is carried from the toes up to the lower fragment of the patella, and a similar bandage is carried from the upper fragment to the upper third of the thigh. In the putting on of either bandage, a fold of strong wire is interwoven so as to leave a loop projecting like a buckle immediately over each fragment of the fractured patella, and as soon as the dressing is sufficiently hard, the fragments of the patella are approximated as near as possible by a piece of strong cord passing between the projecting loops or buckles of wire. In order to obtain the best possible result, a wooden posterior splint should be applied to keep the limb perfectly immovable.

*Fractures of the Femur.*—In fractures of the femur differences of opinion exist as to the propriety of placing all cases in plaster, but, if it is considered advisable, the method is to obtain first of all satisfactory extension and counter-extension, and for this purpose different methods have been practiced. The most convenient for those who have a limited *armamentarium*, is to secure counter-extension by placing a sheet around the body beneath the arms, and securing it to some reliable support. Extension is obtained by carrying a wide strip of adhesive plaster along either side of the leg and attaching it by means of a cord to the pulleys used in reducing dislocations of the hip. When extension and counter-extension have been thus obtained, the limb is covered with a fold of old blanket properly fitted, and the plaster then carried up from the toes to the perinæum, then over and around the pelvis so as to render the extremity perfectly immovable. It is necessary to administer an anæsthetic to obtain complete muscular relaxation —*New York Medical Journal.*

